# Theoretical Analysis of the Process Window for Laser Powder-Bed Fusion for Infrared and Green Lasers Using Rosenthal Approximation

**DOI:** 10.3390/ma19122487

**Published:** 2026-06-10

**Authors:** Vi Ho, Leila Ladani, Jafar Razmi

**Affiliations:** School for Engineering of Matter, Transport and Energy, Ira A. Fulton Schools of Engineering, Arizona State University, Tempe, AZ 85281, USA

**Keywords:** Laser Powder Bed Fusion, copper, near-infrared lasers, green lasers, process window, process map, Rosenthal

## Abstract

Lack of fusion (LOF) is a dominant defect in Laser Powder-Bed Fusion (PBF-LB/M) caused by insufficient overlapping between adjacent melt pools. This study introduces a rapid, first-principles model based on Rosenthal’s analytical solution for a moving point heat source to predict melt pool geometry. Using geometric criteria, the model evaluates whether the melt pool width exceeds the hatching distance and whether the melt pool depth exceeds the layer thickness. Based on these conditions, LOF-based process windows are constructed by plotting laser power against scanning speed and classifying each parameter combination as either LOF or no LOF. The process developed here for constructing LOF process windows can be applied to metallic PBF-LB/M systems. As PBF-LB/M of copper is commonly associated with LOF defects, the approach is examined for pure copper by evaluating a range of laser powers and scanning speeds for both near-infrared (NIR) (1064 nm) and green (515 nm) lasers using copper-specific absorptivity values. The resulting process windows are validated against literature-reported relative density data for pure copper, using high relative density values as indicators of full fusion and lower relative density values reported with LOF characteristics as indicators of lack of fusion. For a 30 µm layer thickness, the predicted LOF boundary agreed with 43 of 46 literature-reported copper PBF-LB/M data points when the data were classified using relative density and reported defect morphology. Sensitivity analysis showed that the agreement changed modestly when the relative-density threshold was reduced from 99% to 98.5% and 98% and that near-boundary classifications were sensitive to the selected absorptivity within the reported NIR range. The agreement supports the use of the framework as a preliminary screening tool for identifying LOF-prone parameter regions. By providing a fast, physics-based screening tool for LOF-limited process windows, this framework offers a computationally efficient alternative to high-fidelity numerical simulations commonly used in PBF-LB/M process development.

## 1. Introduction

### 1.1. Laser Powder-Bed Fusion Challenges

Laser Powder-Bed Fusion (PBF-LB/M) is an additive manufacturing (AM) process in which a high-energy laser scans across a thin layer of metal powder, selectively melting regions according to a digital design. The molten material solidifies forming a dense layer, and the process is repeated layer by layer to build a complete solid part. The stability and morphology of the melt pool are governed by the applied energy input, which defines distinct processing regimes. Laser power, scanning speed, hatching distance, and layer thickness are among the most common parameters used to control this energy input. At low energy input, the melt pool may not become wide or deep enough to ensure sufficient overlap between adjacent scan tracks and successive layers, resulting in lack-of-fusion (LOF) defects [1]. In this regime, unstable melt tracks and discontinuous bead formation may also occur. As energy input increases, the process transitions to stable heat conduction melting, where sufficient overlap between adjacent tracks and layers can be achieved. At excessively high energy input, however, vaporization and recoil pressure can induce deep-penetration or keyhole modes, which are associated with pore formation and process instability [2]. These processing regimes highlight the difficulty of selecting PBF-LB/M parameters. When the energy input is too low, the melt pool may not develop sufficient width or depth to achieve proper overlap, resulting in lack-of-fusion defects. When the energy input is too high, vaporization and recoil pressure can destabilize the melt pool and promote keyhole-related porosity [2]. In addition, the rapid and localized heating and cooling inherent to PBF-LB/M generate steep thermal gradients, which may lead to residual stresses, deformation, and cracking. Process-window development therefore requires balancing these competing mechanisms rather than simply increasing the energy input [3].

### 1.2. Existing Approaches for PBF-LB/M Process-Window Development

Formation of a fully dense, defect-free part requires precise optimization and control of the process. In certain cases, PBF-LB/M becomes especially challenging for metals with high optical reflectivity and/or high thermal conductivity, such as copper, aluminum, and silver [4,5,6]. These materials absorb laser energy inefficiently, and in metals with high thermal conductivity heat is rapidly dissipated away from the melt region, making it difficult to sustain a stable melt pool. Because of these unique physical mechanisms dictating the manufacturing of these materials, studies related to optimization or development of process window for highly conductive and highly reflective metals are scarce.

The optimum level of parameters that could result in a quality build is often referred to as a process window. Multiple approaches have been developed to map process windows for PBF-LB/M, each with distinct advantages and limitations. Experimental process-window mapping, using single-track scans or bulk part builds, is the most direct method, but it is costly and time-intensive due to the need for repeated printing and failure analysis [7]. Statistical optimization approaches based on design of experiments (DoE) and ANOVA have also been used to identify PBF-LB/M parameter combinations that improve relative density and surface quality [8]. However, such approaches still require experimental campaigns to generate fitted relationships, which makes them time- and resource-intensive, and the resulting models are largely correlation-based rather than directly tied to the underlying melt pool physics. In situ imaging methods such as synchrotron X-ray tomography or high-speed thermal cameras provide deeper insight into melt pool dynamics but require specialized equipment and remain impractical for routine parameter optimization [9].

Numerical simulations, particularly finite element (FEM) or finite volume methods (FVM), are widely used to simulate the temperature distribution and melt pool geometry by solving the governing conservation equations of the process, including energy conservation with phase change and, in more comprehensive models, conservation of momentum and mass to capture fluid flow within the melt pool [10,11,12,13]. Numerical models simulate the PBF-LB/M process for a specific set of process parameters and, in more comprehensive cases, also resolve melt pool flow behavior. They therefore provide a detailed description of the process, but each simulation typically corresponds to one parameter set at a time. These models are based on a rigorous set of physical assumptions whose complexity depends on the level of model fidelity. Common assumptions include representing the laser as an idealized moving heat source and treating the powder bed and solid material as continuum regions rather than explicitly resolving individual particles. Depending on model fidelity, additional effects such as surface heat losses due to convection and radiation, temperature- and phase-dependent thermophysical properties, melt pool fluid flow, and melting/solidification behavior may also be incorporated. For example, Mukherjee et al. [10] used a 3D transient FVM model implemented in Fortran to predict melt pool shape and introduced a dimensionless lack-of-fusion number (LF) correlated with experimental void fractions. Johnson et al. [11] developed FEM-based melt pool simulations and trained Gaussian process models to predict simulation outputs across the process space, which were then used to construct defect-based printability maps for Ni-based and high-entropy alloys. Recent efforts have been made to reduce the computational cost of numerical simulations for PBF-LB/M. Foteinopoulos et al. [14] proposed a space-partitioning and dynamic mesh-adaptation approach, in which the computational domain is divided into smaller subdomains that are solved independently while exchanging heat through the subdomain boundaries. This reduced the total simulation time by 74.25%. The maximum temperature differed from the standard model by only 0.16%, although larger local deviations of up to 4.37% were reported during cooling and near the artificial internal boundaries. However, even with this reduction, the simulation still required 10.58 h to complete. While accurate, these high-fidelity methods are complex and demand substantial computational time for each simulation [15].

To reduce complexity, several researchers have developed simplified or semi-empirical models. These models aim to predict melt pool dimensions, such as width and depth, directly from process parameters without resolving the full thermal and fluid flow fields. These include scaling laws based on fitted experimental data to define melt pool geometry as a function of power, speed, and absorptivity [16]. Rubenchik et al. [16] created universal shape functions for melt pool width and depth, but the model only used 316 steel, Inconel and Ti-6Al-4V. Jadhav et al. [17] later applied similar methods to copper, but their approach assumed a constant melt pool diameter equal to the beam diameter—an oversimplification that limits predictive accuracy for overlap and lack of fusion. Other studies have proposed defect-specific thresholds, such as under-melt and balling criteria [18], or keyhole-mode melting based on normalized enthalpy scaling [19], but these models are typically based on fitted scaling relations or simplified defect criteria, so their predictive capability depends on the materials and parameter ranges used in their development. These models rely on a reduced physical description of the process, typically neglecting melt pool flow and evaporation-related effects, representing the melt pool dimensions with simplified geometries, and using scaling relations with reference thermophysical properties instead of full physics resolution.

Analytical models such as the Rosenthal equation [20] offer a physics-based alternative that is both fast and inexpensive. Rosenthal’s solution describes the steady-state temperature distribution around a moving point heat source and can be used to calculate whether the local temperature exceeds the melting point, thereby predicting melt pool shape and overlap. Unlike scaling laws, it requires no fitted data and can be directly applied to any material using known thermal properties. It is particularly well suited for predicting LOF by evaluating whether adjacent tracks will overlap sufficiently both in the lateral and build direction, based on the calculated melt pool width and depth. In this context, porosity is formed from the portion of material left unmelted when neighboring melt pools do not overlap enough. Tang et al. [1] used melt pool dimensions, hatching distance, and layer thickness within a geometry-based framework to predict lack-of-fusion porosity, with melt pool dimensions obtained from the Rosenthal temperature distribution. Another example is Richter et al. [21]. Their framework derives analytical expressions for how much of the material between adjacent tracks and layers is actually melted, extends the treatment to unequal neighboring melt pools, and also considers cases where melt pool width and depth vary from track to track rather than remaining fixed. This allows the model to estimate how melt pool variability changes the predicted porosity.

### 1.3. Scope and Objectives of the Present Study

Tang et al. [1] derived a closed-form approximation from the Rosenthal solution to estimate melt pool width. Using typical PBF-LB/M reference conditions (absorbed power on the order of 100 W, beam speed ≈ 1 m/s, and T_0_ ≈ 303 K), they showed that the Rosenthal equation governing the melt pool boundary can be written as follows:

(1) $$1+\ln\left(r\times \frac{2\pi k\left(t_{m}-t_{0}\right)}{Q\varepsilon }\right)=-\ln\left(r\times \frac{2\pi k\left(t_{m}-t_{0}\right)}{Q\varepsilon }\right)\times \frac{2\alpha }{vr}$$

where r is the distance from the heat source to the melt pool edge at the location of maximum width, k is the thermal conductivity, $t_{m}$ is the melting temperature, $t_{0}$ is the powder-bed temperature, Q is the supplied laser power, ε is the absorptivity, α is the thermal diffusivity, and v is the scanning speed. It should be noted that Equation (1) follows from Rosenthal’s analytical solution to a pure heat conduction equation for a moving point heat source. As such, convection within the melt pool and convective and radiative heat losses are not part of the governing formulation and are outside the scope of the present analytical model.

By numerically solving for typical PBF-LB/M conditions, Tang et al. demonstrated that, for maraging steels, stainless steels, and Ti-6Al-4V, the right-hand side of Equation (1) is negligibly small. This occurs because the ratio 2α/(vr) is much less than 1 for these materials at typical scanning speeds and melt pool dimensions. This is due to two factors: the relatively low thermal diffusivity (α) of these materials and the resulting larger melt pool radius (r). The low diffusivity also allows heat to remain localized near the heat source, increasing r. Consequently, 2α/vr is much less than 1, and the right-hand side of Equation (1) can be neglected. This approximation was validated against experimentally measured melt pool dimensions reported in the literature.

For high-thermal-diffusivity materials, such as copper, the simplification used in previous Rosenthal-based treatments is no longer strictly valid. This is because the right-hand-side term in Equation (1) is not negligible, meaning that the melt pool radius cannot be obtained using the simplified approximation. As shown in Table 1, this term remains meaningfully non-zero for highly conductive metals such as Cu, Ag, Al, and Au under representative PBF-LB/M conditions. Therefore, numerical evaluation of the full Rosenthal-based equation is required for these materials.

The present work aims to identify process parameters that are likely to cause LOF defects. First, Equation (1) is solved numerically to calculate the melt pool dimensions. Then, geometrical overlap criteria are applied to determine whether the calculated melt pool is wide and deep enough to satisfy the hatching distance and layer thickness requirements. Parameter combinations that do not satisfy these criteria are classified as LOF. The final process window presents these classifications on a laser power–scanning speed map, showing the regions where lack of fusion is likely to occur. The proposed framework is therefore intended as a lower-bound LOF screening tool that identifies parameter combinations unlikely to satisfy the minimum melt pool overlap requirements, rather than a complete defect map for all PBF-LB/M instability mechanisms.

Copper was selected as the validation material because it is particularly challenging to process by PBF-LB/M. Its high thermal conductivity and low NIR absorptivity increase the likelihood of incomplete melting and lack-of-fusion defects, making it a valuable test case for evaluating the proposed framework [22]. In this work, LOF process windows for pure copper in PBF-LB/M were constructed using Rosenthal’s analytical solution. The required inputs are material properties, absorptivity, laser power, scanning speed, hatching distance, and layer thickness, and the framework produces melt pool dimensions and LOF process windows.

## 2. Methodology

The primary objective of this study is to construct process windows that identify LOF risks based on geometrical overlap criteria, rather than developing a new thermal model. This section outlines the modeling assumptions, Rosenthal equation derivation, computational steps used to apply Rosenthal’s equation in this context, and the overall analysis workflow of this manuscript.

### 2.1. Assumptions and Simplifications

- Within the melt pool, heat transfer is assumed to be conduction-dominated. Convection within the melt pool, including micro-scale fluid flow effects, and convective and radiative heat losses are therefore neglected. This is consistent with the general understanding that conduction dominates over convection and radiation in PBF-LB/M [23]. In laser melting, the highly localized heat input produces strong temperature gradients along the liquid surface. Because surface tension varies with temperature, these gradients generate stresses that drive liquid-metal flow within the melt pool [24]. This flow can redistribute heat and molten material, reducing the steepness of the local temperature field and altering the melt pool width, depth, and surface morphology, although it is noted that this effect is not substantial [25]. Further, Inclusion of melt pool fluid flow would require extending the model beyond the conduction-based Rosenthal formulation and would eliminate the closed-form analytical structure, leading to significantly increased computational complexity. Such effects are therefore outside the scope of the present first-order analytical framework.
- Thermal conductivity, specific heat, and density were treated as constant and temperature-independent to preserve the analytical structure of the Rosenthal solution and enable rapid evaluation of melt pool dimensions across a broad process-parameter space. This simplification can affect the predicted thermal history because thermophysical properties vary with temperature. For example, Keeley et al. developed a temperature-dependent Rosenthal formulation for Inconel 718 and showed that using constant thermophysical properties could either underpredict or overpredict cooling gradients depending on whether room-temperature or high-temperature conductivity was selected [26]. However, they also reported that melt pool width was accurately predicted in all cases, while the larger discrepancies were mainly associated with cooling gradients and melt pool length. Therefore, although temperature-dependent properties are important for high-fidelity thermal-history or microstructure prediction, the constant-property approximation is considered acceptable in the present work because the model is used as a first-order LOF screening tool based primarily on melt pool width and depth.
- Latent heat of fusion and surface heat losses due to convection and radiation are not directly included in the Rosenthal temperature solution. This follows the classical Rosenthal framework and preserves the analytical structure required for rapid process-window construction. To assess the effect of these neglected terms, their magnitudes were estimated separately relative to the absorbed laser power. The latent heat contribution was estimated from the Rosenthal-predicted melt pool size and the swept molten volume, while convection and radiation were estimated from the surface temperature field using standard heat-flux expressions. Across the evaluated LPBF-relevant conditions, the combined latent heat, convective, and radiative losses reached a theoretical maximum of approximately 2% of the supplied laser power, while most conditions were approximately 0.6% or less. Therefore, these terms are expected to cause only a small quantitative shift in the predicted melt pool boundary and are not expected to change the first-order LOF screening trends. This treatment is also consistent with prior Rosenthal-based LOF modeling by Tang et al. [1], who neglected similar losses while obtaining melt pool width and depth predictions that agreed well with experimental measurements for stainless steel and Ti-6Al-4V.
- The laser is modeled as a point heat source moving at constant velocity across a semi-infinite solid. This approximation treats the absorbed energy as being concentrated near the laser interaction zone and is appropriate for conduction-dominated melting conditions. Within this assumed regime, the resulting process map is intended to evaluate the lower bound condition for LOF avoidance, based on whether the predicted melt pool width and depth satisfy the geometric overlap criteria. The model does not separately classify conduction, transition, or keyhole regimes. This distinction is important because, if keyhole behavior develops, strong vaporization can form a deep and narrow vapor cavity, causing part of the laser energy to be absorbed along the keyhole depth rather than only near the surface. In such cases, previous analytical heat conduction models have noted that line heat sources are more appropriate than point heat sources [27]. Therefore, the present point source formulation should be interpreted as a conduction mode LOF-screening approximation rather than a complete description of all melting regimes.
- Powder porosity, packing density, particle size distribution, powder-layer uniformity, and thermal contact resistance are not explicitly modeled. In a loose powder bed, the effective thermal conductivity, density, heat capacity, and thermal diffusivity can differ from those of consolidated copper because heat must pass through particle–particle contacts and the surrounding gas phase [25]. These effects can influence the local temperature field and therefore shift the predicted melt pool width and depth. In particular, using a lower powder-bed thermal conductivity would reduce conductive heat dissipation away from the laser track, producing a more localized temperature field and potentially predicting larger melt pool dimensions or lower LOF thresholds. However, once melting begins, the molten/solidified copper and underlying substrate strongly affect heat flow, so the effective heat-transfer path is not governed only by the loose powder layer. The Rosenthal solution treats the material as a homogeneous continuum and does not resolve individual particles, gas gaps, powder-layer morphology, or contact resistance. Consistent with prior Rosenthal-based LOF models [1], bulk material properties are therefore used to estimate conductive heat spreading, while powder absorptivity is used to represent laser energy absorption at the powder surface. This is because the model estimates the melt pool boundary for first-pass LOF screening rather than resolving powder-scale heat transfer. Preliminary model checks showed that using an estimated powder-bed conductivity reduced agreement with the literature-based LOF classifications. Bulk copper conductivity was therefore retained in the present calculations.
- The laser input is defined as the product of incident laser power Q and absorptivity. In the present model, absorptivity is treated as an effective constant value for each laser wavelength. In reality, absorptivity depends not only on wavelength but also on powder condition, surface state, temperature, phase, and processing regime. For copper, literature reports show that absorptivity differs between flat solid copper and powder layers under NIR irradiation and that powder-bed absorptivity can vary with powder morphology and processing conditions [22]. During PBF-LB/M, absorptivity may also change as the material transitions from loose powder or consolidated solid to liquid metal and under high-energy conditions (keyhole regime) [28]. Since absorptivity enters the Rosenthal solution through absorbed energy, changes in the absorptivity directly shift the predicted melt pool width, depth, and LOF boundary. However, Tang et al. [1] using a similar Rosenthal model showed that melt pool width is approximately proportional to the square root of absorbed power. This means that uncertainty in absorptivity has a reduced effect on the predicted width. In this work, the absorptivity is treated as a constant and should be interpreted as an effective input parameter for first-order LOF screening rather than a dynamically evolving optical property. The influence of the actual absorptivity value used is evaluated through the absorptivity sensitivity analysis.

### 2.2. Derivation of the Temperature Field and the Melt Pool Size

We begin with the classical three-dimensional transient heat conduction equation in Cartesian coordinates. A global Cartesian coordinate system (x, y, z) is used to describe positions in the build domain, where x is the scan direction, y is the transverse direction, and z is the build direction. Assuming constant material properties, the governing equation is the following [29]:

(2) $$\frac{\partial^{2}t}{{\partial x}^{2}}+\frac{\partial^{2}t}{{\partial y}^{2}}+\frac{\partial^{2}t}{{\partial z}^{2}}=\frac{1}{\alpha }\frac{\partial t}{\partial s}$$

Here, t is the temperature, s is time, and α is the thermal diffusivity of the material. This equation describes how heat spreads over time within a solid due to conduction. To focus on the region around the laser as it moves steadily across the material, we perform a coordinate transformation into a reference frame that moves with the heat source. Let *ξ* = *x* − *v* *s*, where *v* is the constant speed of the heat source [29]. Thus, x denotes the global coordinate, while ξ denotes the coordinate in the beam-fixed reference frame used in the derivation. In this moving frame, the time derivative of temperature t with respect to time s becomes the following:

(3) $$\frac{\partial t}{\partial s}=-v\frac{\partial t}{\partial \xi }$$

Additionally, since ξ replaces x in the moving frame, we have the following:

$$\frac{\partial^{2}t}{{\partial x}^{2}}=\frac{\partial }{\partial x}(\frac{\partial t}{\partial x})$$

$$\Leftrightarrow \;\frac{\partial^{2}t}{{\partial x}^{2}}=\frac{\partial }{\partial x}(\frac{\partial t}{\partial \xi }\frac{\partial \xi }{\partial x})$$

$$\Leftrightarrow \;\frac{\partial^{2}t}{{\partial x}^{2}}=\frac{\partial }{\partial x}(\frac{\partial t}{\partial \xi }*1)$$

$$\Leftrightarrow \;\frac{\partial^{2}t}{{\partial x}^{2}}=\frac{\partial }{\partial x}\frac{\partial t}{\partial \xi }$$

$$\Leftrightarrow \;\frac{\partial^{2}t}{{\partial x}^{2}}=\frac{\partial }{\partial \xi }\frac{\partial t}{\partial x}$$

(4) $$\frac{\partial^{2}t}{{\partial x}^{2}}=\frac{\partial^{2}t}{{\partial \xi }^{2}}$$

Substituting into the original heat equation, we obtain the steady-state form in the moving coordinate system:

(5) $$\frac{\partial^{2}t}{{\partial \xi }^{2}}+\frac{\partial^{2}t}{{\partial y}^{2}}+\frac{\partial^{2}t}{{\partial z}^{2}}=-\frac{v}{\alpha }\frac{\partial t}{\partial \xi }$$

This equation models steady-state heat conduction in the presence of a moving point heat source. To solve it, we assume a temperature solution of the form [29]:

(6) $$t\left(\xi ,y,z\right)=t_{0}\;+e^{\frac{-v\xi }{2\alpha }}*\phi (\xi ,y,z)$$

The exponential term is dimensionless, and the function ϕ (ξ, y, z) has units of temperature. This expression represents the total temperature as a sum of the ambient temperature t_0_ and a decaying exponential multiplied by a shape function ϕ. The exponential term accounts for the asymmetry of temperature distribution in the x direction. Substituting this expression into the heat equation and expanding all terms leads to a simplified equation for ϕ, after factoring out the exponential:

(7) $$\frac{\partial^{2}\phi }{{\partial \xi }^{2}}+\frac{\partial^{2}\phi }{{\partial y}^{2}}+\frac{\partial^{2}\phi }{{\partial z}^{2}}-{\left(\frac{v}{2\alpha }\right)}^{2}\phi =0$$

Assuming the function ϕ is spherically symmetric, we switch to spherical coordinates and define the radial distance from the source as follows:

(8) $$r=\sqrt{\xi^{2}+y^{2}+z^{2}}$$

Letting ϕ = ϕ(R), the Laplacian part of the Equation (7) becomes the following:

(9) $$\nabla^{2}\phi =\frac{d^{2}\phi }{{dR}^{2}}+\frac{2}{R}\frac{d\phi }{dR}$$

Which gives the heat equation as follows:

(10) $$\frac{d^{2}\phi }{{dR}^{2}}+\frac{2}{R}\frac{d\phi }{dR}-{\left(\frac{v}{2\alpha }\right)}^{2}\phi =0$$

Assuming that the solution to this differential equation is ϕ = f(R)/R, simplifying yields a second-order differential equation as follows:

(11) $$f^{\prime \prime }-({\frac{v}{2\alpha })}^{2}f=0$$

which gives the following solution:

(12) $$\phi {=\frac{A}{R}e}^{\frac{-vr}{2\alpha }}$$

The constant A is determined by applying an energy balance. The total heat flux through a spherical surface enclosing the point source must equal the absorbed laser power Q*ε. Using Fourier’s law and assuming symmetry, this results in the following:

(13) $$A=\frac{Q\times \varepsilon }{2\pi k}$$

Substituting this into the solution, we arrive at the final form of the Rosenthal temperature distribution [29]:

(14) $$t=t_{0}\;+\frac{Q\varepsilon }{2\pi kr}e^{-\frac{v(\xi +r)}{2\alpha }}$$

This equation captures the temperature field surrounding a moving point heat source in a semi-infinite body. It shows how the temperature decays both in the direction of laser motion and radially away from the source, providing a physically reasonable approximation of melt pool shape.

**Figure 1:**
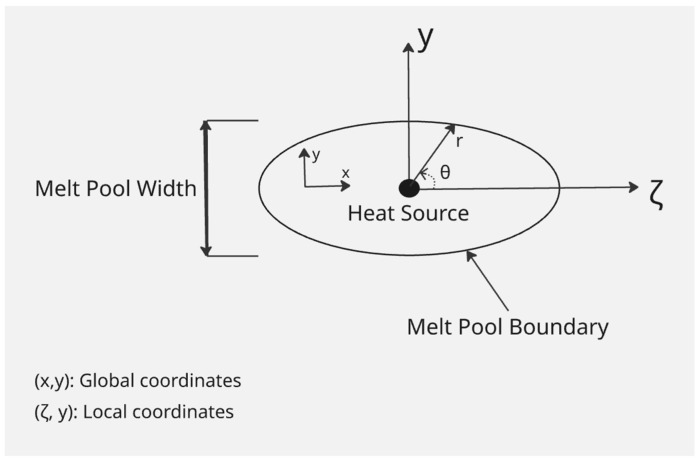
Illustration of the melt pool geometry and the global and local coordinates.

To estimate the width of the melt pool, we focus on the melting isotherm, where the temperature equals the melting point, t = t_m_. Using the Rosenthal temperature field, the lateral distance where this condition is satisfied is determined. Figure 1 shows the geometry used for this derivation at z = 0, which corresponds to the melt pool on the top surface. The goal is to determine the maximum width of the melt pool, which is the point where the melt pool is the widest. To find this, we define $r=\sqrt{\xi^{2}+y^{2}}$ and solve the temperature equation for y as shown in Figure 1, given t = t_m_. By treating the melt pool boundary as a curve, the width is maximum where the slope of the melt pool boundary is zero, dy/dξ = 0. By enforcing this condition, we can derive a compact expression for the melt pool half-width y in terms of a radial distance r, defined as the distance from the heat source to the melting boundary. Following the derivation of Tang et al. [1], we reproduce the key steps here for completeness.

To simplify the temperature distribution expression, we define two key constants:

(15) $$N=\frac{2k\pi (t_{m}\;-t_{0})}{Q\varepsilon }$$

(16) $$M=\frac{v}{2\alpha }$$

We then re-parameterize the spatial coordinates using polar coordinates:

(17) ξ=r∗cosθ

(18) y=r∗sinθ

This substitution allows us to express the melt pool boundary (t = t_m_ isotherm) as a function of the radial distance r and angular coordinate θ:

$$t_{m}=t_{0}+\frac{Q}{2*\pi *k*r}*e^{-\frac{v*\left(\xi +r\right)}{\;2*\alpha }}$$

Substituting Equations (14) and (15) into this equation and taking an ln of both sides, we get the following relationship:

(19) $$\ln\left(N*r\right)+M*r\left(cos\theta +1\right)=0$$

To solve for the radial distance r of the edge of the melt pool from the point source, we need to express the previous equation in terms of constant M, N and the variable r only. In order to replace θ with r, we need to find the relationship dr/dθ. We start by finding where the melt pool reaches its maximum width at the point where its tangent is horizontal, i.e., where the slope of the melt pool boundary satisfies the following:

$$\frac{\mathrm{dy}}{d\xi \;}=0$$

To evaluate this condition, we recognize that both ξ and y are functions of the two variables r and θ. Thus, we compute their total differentials using the chain rule:

(20) $$\frac{d\xi }{d\theta }=cos\theta *\frac{dr}{d\theta }-r*sin\theta$$

(21) $$\frac{dy}{d\theta }=sin\theta *\frac{dr}{d\theta }+r*cos\theta$$

Then, the total derivative becomes the following:

$$\frac{dy}{d\xi }=\frac{dy}{d\theta }\frac{d\theta }{d\xi }$$

(22) $$\Leftrightarrow \;\frac{dy}{d\xi }=\frac{sin\theta *\frac{dr}{d\theta }+r*cos\theta \;}{cos\theta *\frac{dr}{d\theta }-r*sin\theta \;}$$

Setting this to zero leads to the geometric condition for maximum melt pool width:

$$sin\theta *\frac{dr}{d\theta }+r*cos\theta =0$$

(23) $$\Leftrightarrow \frac{dr}{d\theta }=-\frac{cos\theta \;}{sin\theta \;}*r$$

This result expresses how r must vary with θ to satisfy the horizontal tangent condition. We now return to the updated Rosenthal equation and differentiate both sides with respect to θ. This results in the following equation:

$$\frac{1}{r}*\frac{dr}{d\theta }+M\left(\frac{dr}{d\theta }\left(cos\theta +1\right)+r\left(-sin\theta \right)\right)=0$$

(24) $$\Leftrightarrow \;\frac{dr}{d\theta }=\frac{rsin\theta }{\left(\frac{1}{r*M}+\left(cos\theta +1\right)\right)}$$

We now equate the expressions for dr/dθ from the geometric and thermal considerations:

(25) $$\frac{dr}{d\theta }=-\frac{cos\theta \;}{sin\theta \;}*r=\frac{rsin\theta }{\left(\frac{1}{r*M}+\left(cos\theta +1\right)\right)}$$

After some simplification, we can derive the following relationship:

(26) $$\Leftrightarrow \;cos\theta =-\frac{-r*M\;}{1+r*M}$$

Substituting this into Equation (19) we come to the following expression [1]:

(27) $$\Leftrightarrow \;1+\ln\left(N*r\right)=-\frac{ln(r*N)}{r*M}$$

Using r, the melt pool half width y is calculated by the following:

(28) $$y=r*\frac{\sqrt{1+2*r*M}}{1+r*M}$$

It should be noted that for pure copper, t_m_ = 1358 K [30], and this temperature is used throughout the Rosenthal-based calculations to define the melt pool boundary. This choice is suitable for the present conduction-based model because the model predicts the location where the temperature reaches the onset of melting, which provides a simple and physically consistent boundary for estimating melt pool dimensions. In addition, due to the symmetry of the Rosenthal temperature field in the plane perpendicular to the ξ axis, the melt pool boundary is semicircular. As a result, y is the melt pool half width as well as the melt pool depth.

### 2.3. Analytical Framework

We begin with Rosenthal’s three-dimensional steady-state heat conduction solution for a point source moving at constant velocity. The solution yields a temperature field T(x, y, z) that decays spatially from the moving source. The solution is then used to derive a closed-form equation for the dimension of the melt pool (the melt pool width and depth).

In PBF-LB/M, the laser follows a series of parallel scan lines separated by a specified hatching distance *h*. To ensure full fusion between adjacent tracks, each scan line must partially remelt the edge of the previous one. This requirement imposes a geometrical condition: the melt pool width must be at least as wide as the hatching distance. This ensures that no unmelted powder remains between each scan line.

Similarly, from a vertical perspective, each newly deposited layer must fuse with the previous layer, thus leaving no unmelted powder between the layers. This means that the melt pool depth must exceed the layer thickness L. Hence, the geometrical lack-of-fusion (LOF) criterion consists of two conditions:

- Melt pool width ≥ hatching distance.
- Melt pool depth ≥ layer thickness.

Violating either condition is assumed to result in LOF porosity. These geometrical constraints provide a physically meaningful and easily computable threshold for determining viable process parameters.

### 2.4. Process-Window Construction

**Figure 2:**
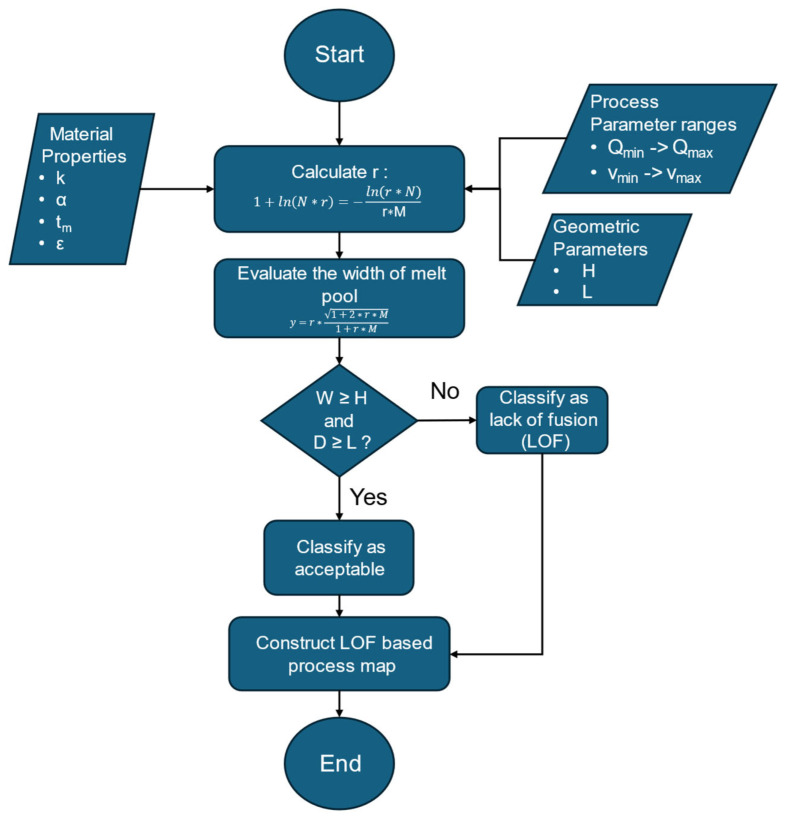
Rosenthal-based workflow for estimating melt pool dimensions and constructing LOF process windows for PBF-LB/M. Here, k is the thermal conductivity, α is the thermal diffusivity, T_m_ is the melting temperature, T_0_ is the initial powder-bed temperature, ε is the laser absorptivity, H is the hatching distance, L is the layer thickness, Q is the laser power, Q_min_ and Q_max_ are the minimum and maximum laser power considered in the process window, v is the scanning speed, v_min_ and v_max_ are the minimum and maximum scanning speed considered in the process window, N = 2πk(t_m_ − t_0_)/(Qε), M = v/(2α), r is the radial distance from the heat source to the melt pool boundary at the point of maximum width, y is the melt pool half width, W is the melt pool width, and D is the melt pool depth.

In this work, process windows (or process windows) refer to plots of laser power versus scanning speed, where each parameter combination is classified based on whether the predicted melt pool geometry satisfies the geometrical overlap criteria for full fusion or results in lack of fusion (LOF). This workflow is demonstrated in Figure 2. Process windows are constructed by evaluating the Rosenthal solution over a predefined grid of laser power and scanning speed combinations. For each parameter set, melt pool dimensions are computed and compared against the geometrical overlap criteria to identify LOF risk.

To identify parameter sets that meet the LOF criteria, we solve Equations (27) and (28) numerically to compute melt pool dimensions for different combinations of laser power and scanning speed. For each parameter set, Equation (28) gives the melt pool half-width y, so the full melt pool width is taken as W = 2y. Since the melt pool boundary is semicircular in the plane perpendicular to the ξ axis due to the symmetry of the problem, the melt pool depth is taken as D = y. The computed melt pool width and depth are then compared with the hatching distance and layer thickness. This is done over the large array of laser power and scanning speed where acceptable laser scan parameters are identified. Scanning speed and powers that result in lack of fusion are marked as the LOF region in this process window. 

An alternate approach to calculating the melt pool dimensions is to graphically measure them after plotting the full temperature field (Equation (14)). However, this would require computing a large number of spatial locations for each parameter set. By contrast, the use of Equation (28) allows direct calculation of the melt pool width, making it better suited for process-window construction across broad parameter spaces.

### 2.5. Material Properties

The material properties of copper used for the process-window construction are listed in Table 2. Because copper absorptivity depends strongly on laser wavelength, separate absorptivity values were used for NIR and green laser conditions. For the NIR case, the reported absorptivity range for untreated copper powder was taken as 0.27–0.33, and the representative baseline value was selected as ε = 0.30.

For the green laser case, the reported absorptivity range was taken as 0.72–0.88, and the representative baseline value was selected as ε = 0.80. These baseline values were used to construct the main process windows, while the absorptivity range was tested in the sensitivity analysis to evaluate how uncertainty in laser absorption affects the predicted LOF boundary.

### 2.6. Analysis Workflow

After establishing the Rosenthal-based LOF criterion and process-window construction procedure, the framework was applied to evaluate PBF-LB/M process-window prediction for copper. Surface temperature fields were calculated for copper and selected engineering alloys under identical laser conditions to illustrate how material properties affect the predicted thermal footprint. Copper melt pool dimensions were evaluated under different laser powers and scanning speeds to examine the influence of process parameters on the calculated melt pool width and depth. LOF process windows were then constructed for pure copper under NIR and green laser conditions using wavelength-specific absorptivity values. The predicted LOF boundaries were subsequently compared with literature-reported copper PBF-LB/M density and defect data, as described in the following validation section.

### 2.7. Literature Validation

LOF defects introduce unmelted regions and porosity, and they are associated with reduced relative density. Therefore, literature-reported relative density was used as a proxy for fusion quality. To validate the predictive accuracy of the LOF process window, we overlaid literature-reported PBF-LB/M parameter sets onto our model for a 30 μm layer thickness. Each data point is categorized as follows:

- No lack of fusion: This represents nearly full density with minimal or no LOF porosity. These points are treated as cases where sufficient melt pool formation and overlap were achieved. A 99% relative-density threshold was selected as a practical and conservative criterion for “fully fused” material because the collected literature data are not consistently reported with 0.1% precision, and many values are instead reported as whole-number percentages, approximate values, or ranges. Further, literature reports indicate that LOF pores are rare but may still occasionally be observed at near 99% relative density, making 99% a reasonable threshold for separating near-full fusion from lower-density LOF cases. This threshold is also consistent with the use of densities at or above 99% as indicators of near-bulk material quality.
- Lack of fusion: This represents data points with porosity caused by incomplete melting LOF. A data point is classified as lack of fusion (LOF) if the reported relative density is ≤99% and the original study reports the presence of lack of fusion or microstructural features consistent with LOF. These features include irregular or elongated pores with the presence of partially melted or unmelted powder.
- Excluded: data points are excluded from this study if the relative density is below 99% but the reported defects are attributed to mechanisms other than LOF (e.g., keyhole porosity, balling, or sputtering), or the microstructural evidence is not provided, and defect type cannot be determined.

Each literature data point was then compared with the LOF boundary corresponding to its reported hatching distance. Points above the corresponding hatch-spacing boundary were treated as model-predicted no LOF, while points below the boundary were treated as model-predicted LOF. Agreement was counted when the literature-based classification matched the model prediction. Detailed information regarding the data points used is provided in Appendix A.

### 2.8. Sensitivity Analysis

This sensitivity analysis evaluates whether the main conclusions of the LOF process-window validation remain stable when three modeling assumptions are varied: absorptivity, relative-density threshold, and the temperature at which the thermophysical properties of copper are evaluated.

The sensitivity analysis was performed to evaluate whether the literature-based validation remained stable under reasonable variations in model inputs and classification criteria. Three factors were varied: laser absorptivity, the relative-density threshold used to classify literature data, and the thermophysical-property set used in the Rosenthal calculation. For the absorptivity study, the NIR absorptivity of untreated copper powder was varied over the literature-reported range of 0.27–0.33. For the density-threshold study, the baseline threshold of 99% was reduced to 98.5% and 98% to assess whether the agreement rate was sensitive to the selected classification threshold. A lower bound of 98% was selected because polished cross-sections reported in the literature at approximately this density already show clear LOF porosity. For the thermophysical-property study, copper thermal conductivity, density, specific heat capacity, and thermal diffusivity varied over the solid-temperature range up to 1000 °C, using temperature-dependent values from Mills et al. The agreement rate was recalculated for each sensitivity case using the same literature-validation procedure described above.

### 2.9. Computational Efficiency Assessment

The computational efficiency of the proposed framework was evaluated by recording the runtime required to construct the LOF process windows. The MATLAB (R2025b) implementation was used to evaluate a grid of 10,000 laser power–scanning speed combinations across three hatching distances. Runtime was recorded separately for the process-window calculation and for the total script execution, including plotting. The calculations were performed in a standard desktop MATLAB environment on a computer equipped with an AMD Ryzen 7 processor and 16 GB of RAM.

## 3. Results

### Comparison of Surface Temperature Fields

We can visualize the melt pool shape by numerically plotting the isotherm equal to the melting temperature using Equation (13). Using MATLAB, x, y, and z coordinate values were substituted into Equation (13) to acquire the temperature of each coordinate. These points were then plotted on a map and color-coded based on their temperature to create a temperature distribution map. For this specific purpose, the Rosenthal temperature distribution was used instead of Equation (27), as it provides a more direct way to calculate the temperature field from the process parameters. Each region is colored according to the outer boundary isotherm, and discrete temperature levels are displayed instead of a continuous gradient to make these regions clearer. The reference temperature of 300 K represents the ambient powder-bed temperature [38]. For copper, H13 steel, AlSi10Mg, and Ti-6Al-4V, the temperature fields were plotted from 300 K up to their melting temperatures (1300 K, 1427 K, 590 K, and 1660 K, respectively [39,40,41]). The plot shows ten evenly spaced “isotherm” regions for visualization purposes. The absorptivity used in the simulation corresponds to values at NIR wavelengths [42]: 0.30 for copper [22,35,36,37], 0.38 for H13 steel [43], 0.65 for AlSi10Mg [42], and 0.6 for Ti-6Al-4V [44].

**Figure 3:**
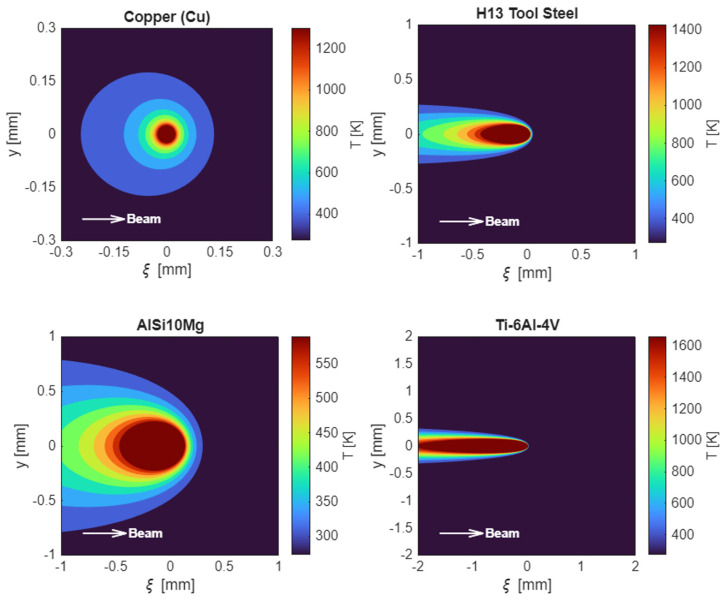
Surface temperature distribution for copper, H13 steel, AlSi10Mg, and Ti-6Al-4V for a PBF-LB/M process using a laser power of 200 W and a scanning speed of 500 mm/s.

Figure 3 shows the surface temperature distributions for different materials subjected to the same laser conditions (200 W, 500 mm/s), computed using Rosenthal’s analytical model. Although the laser power and scanning speed are held constant, the resulting isotherms vary significantly across the materials. Copper and AlSi10Mg exhibit more rounded, compact temperature distributions, whereas H13 tool steel and Ti-6Al-4V show more elongated, oval-shaped isotherms stretched in the direction of laser travel. This trend is most evident in the melt pool regions of each material.

This difference in isotherm shape arises from how rapidly thermal energy diffuses into each material relative to the scanning speed. Because absorptivity, thermal conductivity, and heat capacity differ from one material to another, the heat spreads differently under the same laser conditions. Under typical PBF-LB/M conditions, copper and AlSi10Mg diffuse heat rapidly enough relative to the laser motion that the temperature field spreads laterally as well as along the scan direction, producing more compact and rounded isotherms. In contrast, Ti-6Al-4V and H13 steel diffuse heat less rapidly relative to the scanning speed, so the moving heat source carries the hot region forward faster than the heat can spread sideways, resulting in more elongated isotherms stretched in the direction of travel.

This qualitative trend has been reported in single-track laser melting experiments in the literature. Single-track studies on aluminum alloys and copper consistently show compact, rounded trailing melt-track tails, whereas steels and Ti-6Al-4V exhibit elongated, pointed tails aligned with the scan direction [45,46,47,48]. In addition, in situ surface temperature measurements during PBF-LB/M provide experimental evidence of asymmetric thermal footprints consistent with these trends. High-speed NIR thermography measurements of Ti-6Al-4V show a strongly elongated surface hot zone aligned with the scan direction, characterized by a compressed leading edge and a long trailing thermal tail [49]. This qualitative asymmetry is consistent with the Rosenthal-based temperature fields predicted for low-thermal-conductivity materials. Similar in situ surface temperature measurements for stainless steels also reveal elongated thermal footprints with a trailing tail opposite the scan direction [50]. The degree of elongation observed experimentally is less pronounced than in Ti-6Al-4V, which is consistent with the intermediate thermal properties of steels and with the Rosenthal-based temperature field predicted for H13 tool steel in Figure 3. Although these experimental measurements capture fully transient melt pool dynamics that are not modeled here, they provide qualitative experimental support for the material-dependent thermal trends predicted by the Rosenthal-based temperature fields.

## 4. Process Windows for Lack of Fusion in PBF-LB/M of Pure Copper

### 4.1. LOF Process Windows for Near-Infrared and Green Lasers

Most commercial PBF-LB/M systems use NIR fiber lasers operating at wavelengths between 1060 and 1070 nm. While these lasers are effective for many engineering alloys, they are poorly suited for processing pure copper due to the material’s low absorptivity at NIR wavelengths. In the powder state, copper absorbs only 3–5% of the incident laser energy, with the remainder reflected from the surface. This results in a significant reduction in effective heat input, limiting the ability of the laser to generate and sustain a melt pool. Green lasers on the other hand, operating near 515 nm, interact with copper much more efficiently. The reported powder absorptivity exceeds 70% and can approach 88%, depending on particle morphology and composition [35,37], leading to improved energy coupling. These differences in absorptivity are directly reflected in the predicted process windows.

**Figure 4:**
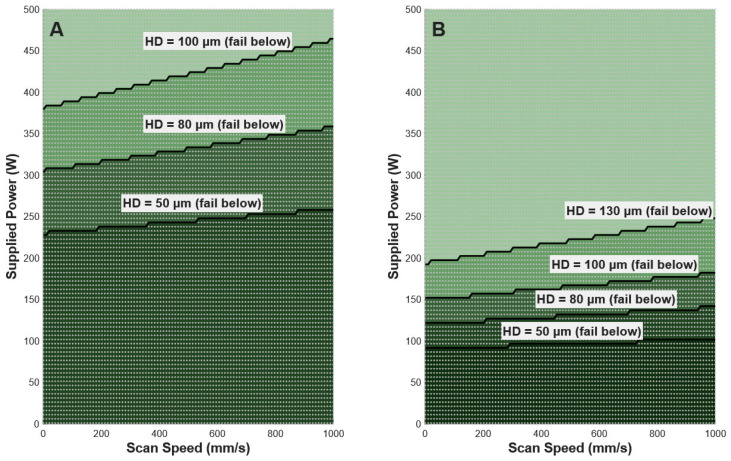
Process window for the PBF-LB/M of copper at a layer thickness of 30 μm for NIR laser (**A**) and green laser (**B**).

Figure 4 shows the resulting process windows. For each hatching distance, the corresponding line marks the boundary between parameter combinations predicted to produce lack of fusion (LOF) and those expected to provide enough melt pool overlap for full fusion. Parameter sets below a given line are predicted to fall in the LOF region, while those above it are expected to avoid LOF. As the hatching distance increases, the melt pool must become wider to maintain overlap between adjacent scan tracks. This makes the fusion requirement more demanding and shifts the LOF boundary toward higher laser powers. Even a modest increase in hatching distance therefore requires a noticeable increase in power to avoid LOF.

For NIR PBF-LB/M, the acceptable region is strongly limited by laser power. At low powers, even a small hatching distance fails to meet the LOF criterion. Increasing power expands the viable region, whereas scanning speed has a much weaker influence. Beyond moderate values, changes in scanning speed produce only small variations in melt pool width. Increasing the hatching distance shifts the LOF boundary rapidly toward higher powers, narrowing the process window and pushing the minimum required power above roughly 400 W for typical spacings.

The green laser case shows much more compact LOF regions in the process window. Because copper absorbs green laser light much more efficiently, a larger fraction of the incident laser power is deposited into the powder bed. For the same nominal laser power, this results in a larger melt pool than in the NIR case. Consequently, full fusion can be achieved at much lower laser powers over a broader range of scanning speeds. Even at large hatching distances, such as 130 µm, the LOF boundary remains near 200–300 W, and for smaller spacings, such as 80 µm, nearly the entire modeled power–speed space satisfies the LOF criterion. In contrast to the NIR case, increasing hatching distance produces only a modest shift in the LOF boundary, indicating a much more robust process window.

**Figure 5:**
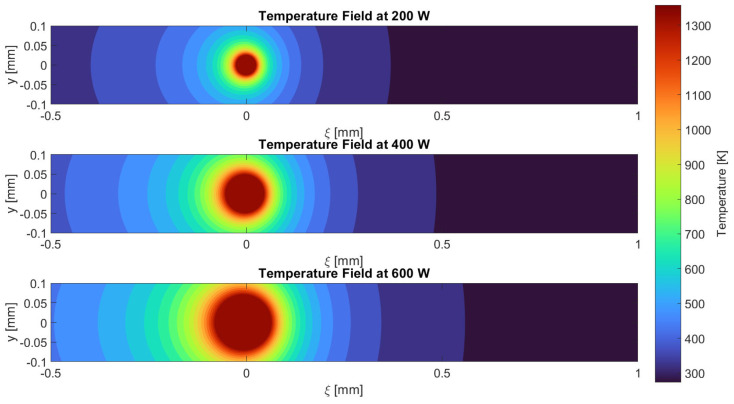
Melt pool size comparisons for PBF-LB/M on copper with laser powers of 200 W, 400 W, and 600 W and a scanning speed of 400 mm/s. The inputs were copper’s thermal conductivity, a fixed scanning speed of 400 mm/s, an absorptivity of 0.35, and laser powers of 200 W, 400 W, and 600 W.

Overall, these process windows suggest that laser power has a stronger influence on melt pool size than scanning speed. This trend is examined in the following section. To show how laser power affects melt pool size, Figure 5 was created by calculating the temperature at each point in a cross-sectional grid using Equation (13), which models heat from a moving laser. For each case, the plot shows how heat spreads out from the laser. The innermost region in each plot corresponds to 1358 K, representing the boundary of the melt pool. Although the center is hotter, this region is color-coded based on the temperature at its boundary to clearly show the melt pool shape. All other regions use the same color scale to allow better visualization.

As shown in Figure 5, the melt pool size increases significantly with laser power. At 200 W, the melt pool diameter is just 50 µm, making it insufficient to satisfy the LOF criterion for any of the typical hatching distances considered (50, 70, or 100 μm). At 400 W, the melt pool expands to 97 µm in width, which is adequate for hatching distances of 50 and 70 μm but still falls short for 100 μm. When the power is increased to 600 W, the melt pool diameter exceeds 141 µm, meeting the LOF requirement for all three hatching distances. These results align with the process window in Figure 4, where higher powers are required to achieve acceptable fusion at larger hatching distances. The visualizations reinforce how strongly melt pool geometry—and thus process viability—is governed by laser power in NIR PBF-LB/M of copper.

**Figure 6:**
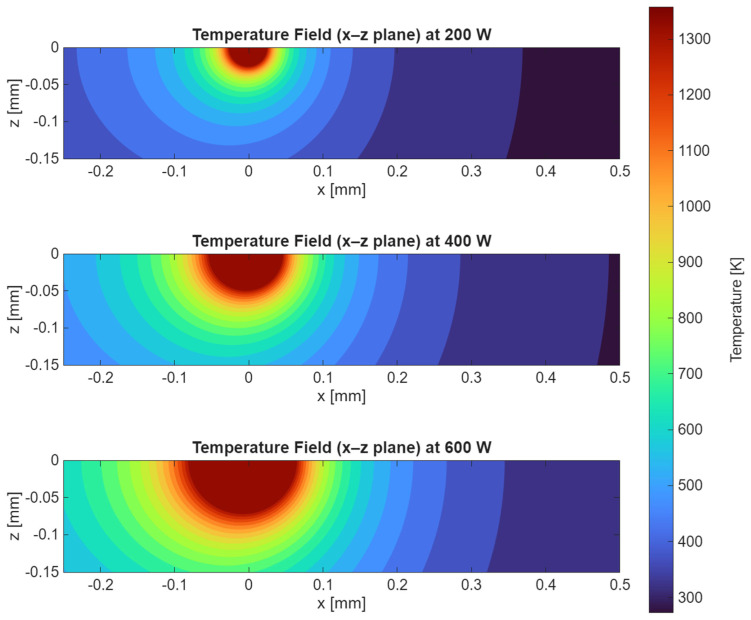
Melt pool depth comparisons for PBF-LB/M of copper with laser powers of 200 W, 400 W, and 600 W and a scanning speed of 400 mm/s.

Similarly, Figure 6 shows the subsurface temperature field and melt pool depth for laser powers of 200, 400, and 600 W at a fixed scanning speed of 400 mm/s. The melt pool depth is equal to half of the melt pool width shown in Figure 5 for the equivalent cases considered here. This is because the temperature field produces a semispherical melt pool cross-section in the y–z plane, as implied by Equation (14). In reality, this assumption is not always correct, especially in the keyhole regime, where the melt pool depth can be significantly larger than the semispherical prediction [17]. In such cases, the depth–width relationship used here becomes conservative: the hatch-distance criterion remains correctly evaluated, while the layer thickness condition may be overly restrictive. The parameter sets excluded under this assumption correspond to cases where the layer thickness is much larger than half of the hatching distance, a regime that is rarely reported in the PBF-LB/M literature; therefore, the process window remains a reasonable and practically relevant screening tool.

**Figure 7:**
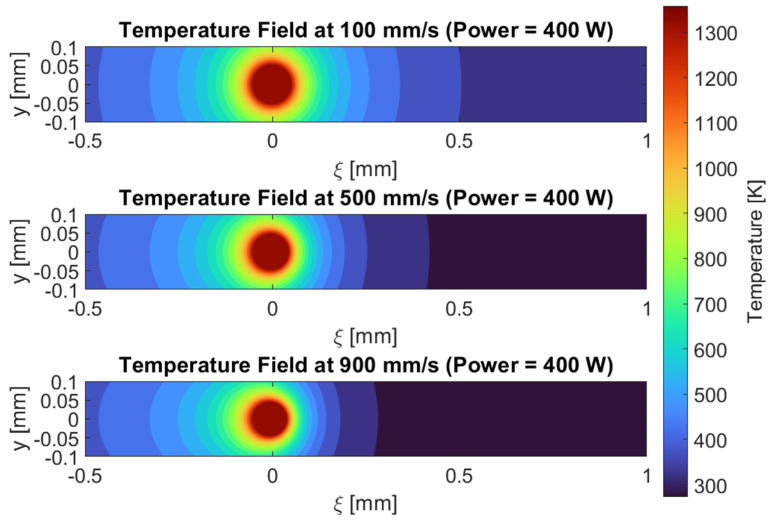
Melt pool size comparisons for PBF-LB/M of copper with a laser power of 400 W and scanning speeds of 100 mm/s, 500 mm/s, and 900 mm/s.

The scanning speed also has an impact on the size of the melt pool. However, its impact is not as profound as laser power. To illustrate this, similar to Figure 5, Figure 7 is constructed to visualize the effects of scanning speed on the melt pool diameter by varying the scanning speed while holding the laser power constant at 400 W.

At a constant laser power of 400 W, variations in scanning speed produce only modest changes in melt pool geometry. The calculated melt pool widths are approximately 102 μm at 100 mm/s, 95 μm at 500 mm/s, and 90 μm at 900 mm/s. Although higher scanning speeds reduce the interaction time between the laser and the material, the overall effect on melt pool width is minor compared with that of laser power. Therefore, the process window for NIR PBF-LB/M of copper is primarily governed by laser power, while scanning speed serves as a secondary parameter with limited impact on achieving full fusion.

### 4.2. Literature-Based Validation of the LOF Process Window

**Figure 8:**
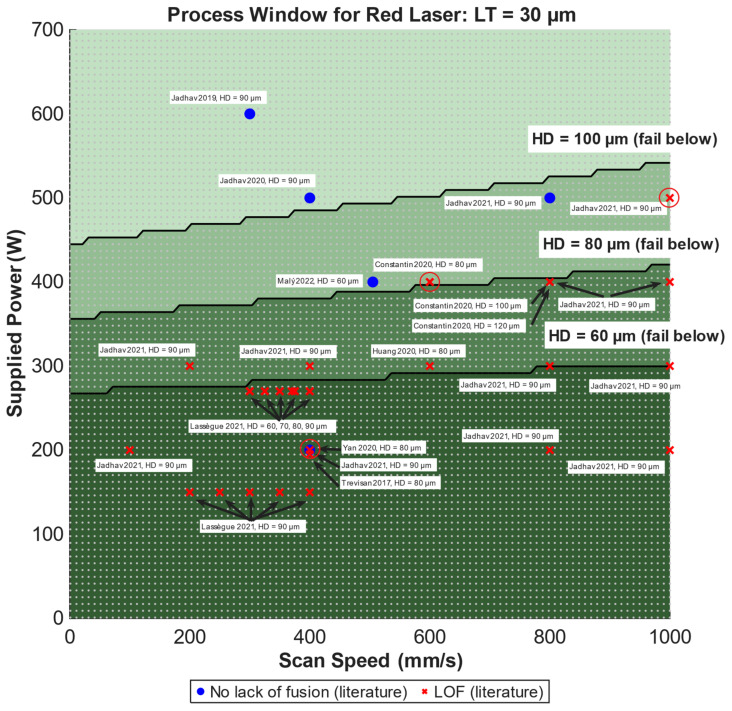
Process window for PBF-LB/M of copper (30 μm layer thickness) overlaid with literature-reported parameter sets. Data in disagreement with the process window are circled in red [17,36,51,52,53,54,55,56,57].

Figure 8 shows the process window for a layer thickness of 30 μm with literature-reported data points overlaid using the classification described above. Each data point is labeled by its hatching distance (HD) and evaluated relative to the LOF boundary corresponding to that value. Parameter combinations above the relevant HD boundary are predicted to provide sufficient melt pool overlap and avoid LOF, whereas combinations below the boundary are predicted to fall in the LOF region.

To illustrate this comparison, the data point labeled “Jadhav 2019, HD = 90 μm” corresponds to a laser power of 600 W and a scanning speed of 300 mm/s. This point is classified as no LOF based on the literature because its reported relative density is above 99%. In the process window, this parameter combination also lies above the HD = 90 μm boundary, indicating that the model also predicts no LOF. Therefore, the experimental classification and the model prediction agree for this case. The same procedure was applied to all literature data points.

Using this approach, 93% of the data points agree with the process-window prediction, while three data points do not. In general, data points classified as no LOF lie above their corresponding HD boundary, whereas those classified as LOF lie below it. The three disagreement cases do not show a clear common trend. These isolated points may reflect additional process-dependent factors not included in the present model. Two of these disagreement points (Constantin et al. (2020) [52] and Jadhav et al. (2021) [17]) are located very close to the predicted LOF boundary. For points this close to the boundary, small differences between the reported process parameters and the actual printing conditions could change the classification. For example, the delivered laser power may differ slightly from the nominal setting, and the actual powder-bed absorptivity may vary with powder condition, packing, and surface state. Therefore, these cases are better interpreted as borderline disagreements rather than clear failures of the process-window model. Overall, the comparison indicates that the Rosenthal-based process window provides a good first-order prediction of LOF as a function of laser power, scanning speed, hatching distance, and layer thickness.

### 4.3. Sensitivity Analysis

The absorptivity sensitivity analysis is summarized in Table 3. The agreement remains unchanged at 43/46 for absorptivity values of 0.27 and 0.30 but decreases to 38/46 when an absorptivity of 0.33 is used.

The reduction in agreement is associated with five data points reported by Lassègue et al. [36], all of which still exhibit LOF defects even though they fall above the predicted boundary. All five occur at a relatively low laser power of 270 W and small hatching distance of 60 μm, indicating that the additional disagreement is confined to the low-power regime rather than being broadly distributed across the process window. It should also be noted that these data points lie very close to the predicted LOF boundary, so only a modest shift in the boundary is sufficient to change their classification. Lassègue et al. [36] attributed this behavior to the combined effect of low laser power and small hatching distance. As the hatching distance decreases, a larger portion of the melt pool comes into contact with previously solidified copper tracks. Because solid copper rapidly conducts heat away from the molten region, this additional heat loss can reduce effective remelting and weaken overlap between adjacent tracks, thereby promoting lack-of-fusion defects.

The density-threshold sensitivity analysis is summarized in Table 4. The density-threshold sensitivity analysis shows only a small reduction in agreement as the threshold is lowered from 99% to 98.5% and 98%. The agreement decreases from 43/46 at 99% to 42/46 at 98.5% and 41/46 at 98%. This indicates that the overall validation outcome is not highly sensitive to modest changes in the selected density threshold. Among the tested thresholds, 99% gives the highest agreement and is therefore retained as the baseline criterion. This supports the use of 99% as a reasonable and conservative threshold for distinguishing nearly fully fused data points from those more likely to contain LOF in the present validation framework.

The thermophysical-property sensitivity check evaluated whether changing the copper property set over the solid-temperature range up to 1000 °C affected the literature-based validation. As temperature increased, the thermal conductivity and thermal diffusivity of copper decreased, causing the predicted LOF boundaries to shift slightly toward lower laser powers. The agreement remained 43/46 when the properties were evaluated below approximately 200 °C. Above this range, the agreement decreased to 38/46 because the HD = 60 µm boundary shifted below five Lassegue et al. [36] data points at 270 W and HD = 60 µm. These data points are located close to the predicted LOF boundary; therefore, a small boundary shift is sufficient to change their classification. This indicates that the model is stable across a broad thermophysical-property range, but near-boundary points remain sensitive to small shifts in the predicted LOF boundary. The highest agreement was obtained when thermophysical properties were evaluated at lower temperatures.

Overall, the sensitivity analysis shows that the agreement with the literature data remains relatively stable under reasonable variations in absorptivity, density threshold, and thermophysical properties. Changes in absorptivity and thermophysical properties mainly affect data points associated with low powers, small hatching distances that are located close to the predicted LOF boundary. The density-threshold sensitivity also shows only a modest change in agreement when the threshold is reduced from 99% to 98.5% and 98%. Therefore, the main process-window trend remains stable, while borderline data points remain sensitive to small shifts in the predicted boundary. Overall, this supports the use of the proposed framework as a first-pass LOF screening tool.

### 4.4. Competitive Advantage of the Proposed Framework

The proposed framework provides a physics-based and computationally efficient approach for identifying lack-of-fusion boundaries in PBF-LB/M. Unlike purely empirical process windows, it is derived from Rosenthal’s analytical solution and evaluates fusion using geometric overlap criteria based on melt pool width and depth. This allows the method to be applied using known material properties and process parameters without requiring fitted data for each new case. Experimental process-window development requires repeated builds and post-process defect analysis, while high-fidelity FEM- and FVM-based simulations generally require the solution of the full coupled thermal and fluid-flow problem for each parameter set. By contrast, the present method uses a direct physical basis through the Rosenthal temperature field while remaining simple enough for rapid evaluation over broad power–speed ranges.

From a practical standpoint, the framework is most useful as a lower-bound LOF screening tool. By identifying parameter sets that do not satisfy the geometric overlap requirements for melt pool width and depth, it allows clearly unviable conditions to be excluded before experimental trials or higher-fidelity simulations. This reduces the parameter space that must be explored using more expensive approaches and provides fast preliminary guidance for PBF-LB/M parameter selection.

The practical efficiency of the framework is reflected in the present implementation. For the grid used in this study, the MATLAB code evaluated 10,000 power–speed combinations across three hatching distances. The process-window calculation itself took 81.17 s, and the total script runtime, including plotting, was 85.09 s. These calculations were performed in a standard desktop MATLAB environment on a system equipped with an AMD Ryzen 7 processor and 16 GB of RAM. This level of computational cost makes the framework suitable for rapid preliminary LOF assessment.

## 5. Discussion

This study presents a predictive model for identifying LOF failure regimes in PBF-LB/M, using a temperature-based analytical framework derived from the Rosenthal equation. By relating the melt pool width to input energy, layer thickness, and hatching distance, the model defines threshold curves below which LOF is likely to occur due to insufficient overlap. When overlaid with literature-reported process parameters and relative density outcomes, the model shows strong agreement. This confirms the model’s utility in estimating the minimum conditions necessary to ensure conduction-mode melting and avoid LOF defects.

Assessment of the process window for copper shows that laser power is more dominant than scanning speed in controlling melt pool size within the ranges of commonly used PBF-LB/M parameters. Increasing laser power leads to substantial expansion of the melt pool width, whereas variations in scanning speed produce relatively small changes. Generally, reducing scanning speed cannot compensate for insufficient power, as rapid heat conduction limits the benefit of extended interaction time. As a result, power is the primary parameter governing the onset of LOF in NIR PBF-LB/M of copper.

Further, the green laser case exhibits a broader and more robust processing window, with minimal sensitivity to hatching distance or scanning speed. This reduces the dependence of melt pool geometry on precise parameter tuning and explains the improved performance reported in green laser PBF-LB/M studies.

The present model predicts whether the calculated melt pool width and depth are sufficient to satisfy the hatching distance and layer thickness requirements. From a practical standpoint, the present model should be interpreted as a lower-bound LOF screening criterion for PBF-LB/M parameter selection. While LOF is a dominant failure mode in low-energy regimes, it is not the only mechanism that determines part quality. A more complete PBF-LB/M process window would include both lower-bound and upper-bound defect limits. The lower-bound limit corresponds to insufficient energy input, where the melt pool width or depth is insufficient to satisfy the hatch-spacing and layer thickness overlap criteria. This lower-bound LOF regime is captured by the present Rosenthal-based framework. However, parameter combinations above this lower bound are not necessarily defect-free, because other defect mechanisms can occur under unstable or excessive-energy conditions. Balling can occur when insufficient energy input lowers melt pool fluidity and causes elongated melt tracks to break into irregular bead-like features; it can also occur at excessive scan speed when melt pool instability disrupts continuous track formation [51,58,59]. At higher energy input, overfusion and keyhole porosity may occur as vaporization, recoil pressure, and Marangoni-driven flow destabilize the melt pool. As the melt pool deepens and collapses, gas can become trapped, forming keyhole pores that reduce relative density [31,60,61]. Spatter or sputtering may also occur under high-energy conditions when vapor recoil and melt pool flow eject molten material from the pool, deteriorating surface quality and local track stability [31,60]. Delamination, curling, and cracking may arise from insufficient interlayer bonding or from high thermal gradients that generate residual stresses, uneven expansion/contraction, and layer separation. This can occur at both extremes of the process window [52,62,63,64]. Therefore, future work could combine the present LOF boundary with additional defect-specific criteria to identify additional regions of the process window that are likely to produce balling, keyholing, spatter/sputtering, delamination, or cracking.

Future versions of this model could be expanded to incorporate additional physics beyond pure conduction. For instance, thermal conductivity and laser absorptivity could be treated as temperature-dependent, improving the accuracy of energy transport predictions. Phase change phenomena, such as latent heat and melting front dynamics, could be added to better model the actual solid-to-liquid transition. Convection-driven flow within the melt pool—currently neglected in the Rosenthal solution—could also be considered to account for Marangoni effects and momentum-driven mixing. Furthermore, melt pool dynamics such as surface deformation, recoil pressure, and keyhole formation could be integrated through either semi-empirical corrections or coupling with fluid dynamics simulations. Such refinements would enable the model to describe not only LOF boundaries but also the full stability envelope of copper PBF-LB/M processing.

Overall, this work contributes a physics-based framework for understanding the geometrical and thermal limits of stable PBF-LB/M processing. It demonstrates that a relatively simple analytical model, rooted in classical heat transfer, can yield actionable insight into parameter selection and defect avoidance, especially when paired with rigorous literature validation.

## 6. Conclusions

This study developed a geometry-based framework derived from Rosenthal’s temperature distribution to estimate melt pool dimensions and construct first-pass LOF process windows for PBF-LB/M of copper. By applying minimum overlap criteria based on hatching distance and layer thickness, the framework identifies parameter regions where insufficient melt pool width or depth may lead to lack-of-fusion defects.

The results show that, within the assumptions of the Rosenthal-based model, larger hatching distance and layer thicknesses require higher laser power to maintain melt pool overlap. The calculated melt pool dimensions also show that laser power has a stronger effect than scanning speed for the copper parameter ranges evaluated in this study.

The predicted LOF boundaries were compared with literature-reported copper PBF-LB/M data and showed good first-order agreement with reported relative density and defect observations. The framework is shown to be a good tool for preliminary LOF screening. It does not capture temperature-dependent material behavior, phase change effects, convection-driven melt pool flow, or defect mechanisms such as keyholing, balling, and delamination. Future work may incorporate these effects and extend the framework to a more complete defect map.

## Figures and Tables

**Table 1:** Magnitude of the non-negligible term in Equation (1) for highly conductive metals.

Material	Right-Hand-Side Term in Equation (1)
Cu	0.992
Ag	0.997
Al	0.973
Au	0.996

**Table 2:** Material constants for copper.

Property	Symbol	Value	Unit
Thermal conductivity	k	400 [31]	W/m·K
Density	ρ	8933 [30]	kg/m^3^
Specific heat capacity	c_p_	385 [30]	J/kg·K
Thermal diffusivity	α	1.16 × 10^−4^	m^2^/s
Melting temperature	T_m_	1358 [30]	K
Ambient temperature	T_0_	303 [32,33,34]	K
Laser absorptivity (NIR)	ε	0.27–0.33 [22,35,36,37]	N/A
Laser absorptivity (green)	ε	0.72–0.88 [22]	N/A

**Table 3:** Absorptivity sensitivity.

#	Absorptivity	Agreement Rate
1	0.27	43/46
2	0.30	43/46
3	0.33	38/46

**Table 4:** Density-threshold sensitivity.

#	Relative Density Threshold (%)	Agreement Rate
1	99	43/46
2	98.5	42/46
3	98	41/46

## Data Availability

The original contributions presented in this study are included in the article. Further inquiries can be directed to the corresponding authors.

## Appendix A

**Table A1:** Literature Data Used for LOF Process-Window Validation.

#	Density (%)	Classification	Reference	Power	Speed	HD	LT
1	99.9	No LOF	Malý et al., 2022 [53]	400	500	60	30
2	99.5	No LOF	Jadhav et al., 2019 [54]	600	300	90	30
3	<95	LOF	Constantin et al., 2020 [52]	40	600	120	30
4	<95	LOF	Constantin et al., 2020 [52]	40	600	10	30
5	<95	LOF	Constantin et al., 2020 [52]	400	600	80	30
6	86	LOF	Lassègue et al., 2021 [36]	270	300	90	30
7	84	LOF	Lassègue et al., 2021 [36]	270	325	90	30
8	83	LOF	Lassègue et al., 2021 [36]	270	370	90	30
9	83	LOF	Lassègue et al., 2021 [36]	270	350	90	30
10	83	LOF	Lassègue et al., 2021 [36]	270	300	90	30
11	82	LOF	Lassègue et al., 2021 [36]	270	400	80	30
12	82	LOF	Lassègue et al., 2021 [36]	270	375	80	30
13	82	LOF	Lassègue et al., 2021 [36]	270	350	80	30
14	82	LOF	Lassègue et al., 2021 [36]	270	325	80	30
15	81	LOF	Lassègue et al., 2021 [36]	270	300	80	30
16	81	LOF	Lassègue et al., 2021 [36]	270	400	70	30
17	81	LOF	Lassègue et al., 2021 [36]	270	375	70	30
18	81	LOF	Lassègue et al., 2021 [36]	270	350	70	30
19	81	LOF	Lassègue et al., 2021 [36]	270	325	70	30
20	81	LOF	Lassègue et al., 2021 [36]	270	300	70	30
21	81	LOF	Lassègue et al., 2021 [36]	270	400	60	30
22	81	LOF	Lassègue et al., 2021 [36]	270	375	60	30
23	81	LOF	Lassègue et al., 2021 [36]	270	350	60	30
24	81	LOF	Lassègue et al., 2021 [36]	270	325	60	30
25	81	LOF	Lassègue et al., 2021 [36]	270	300	60	30
26	80	LOF	Lassègue et al., 2021 [36]	150	300	90	30
27	80	LOF	Lassègue et al., 2021 [36]	150	250	90	30
28	80	LOF	Lassègue et al., 2021 [36]	150	200	90	30
29	79	LOF	Lassègue et al., 2021 [36]	150	400	90	30
30	79	LOF	Lassègue et al., 2021 [36]	150	350	90	30
31	99.3	No LOF	Jadhav et al., 2021 [17]	500	800	90	30
32	98.2	LOF	Jadhav et al., 2021 [17]	400	800	90	30
33	97.6	LOF	Jadhav et al., 2021 [17]	30	200	90	30
34	96.3	LOF	Jadhav et al., 2021 [17]	300	400	90	30
35	92.9	LOF	Jadhav et al., 2021 [17]	40	1000	90	30
36	92.6	LOF	Jadhav et al., 2021 [17]	500	1000	90	30
37	91.9	LOF	Jadhav et al., 2021 [17]	300	800	90	30
38	90.6	LOF	Jadhav et al., 2021 [17]	200	1000	90	30
39	89.7	LOF	Jadhav et al., 2021 [17]	200	400	90	30
40	89.1	LOF	Jadhav et al., 2021 [17]	200	100	90	30
41	89	LOF	Jadhav et al., 2021 [17]	300	1000	90	30
42	87.8	LOF	Jadhav et al., 2021 [17]	200	800	90	30
43	99	No LOF	Jadhav et al., 2020 [55]	500	400	90	30
44	99	No LOF	Yan et al., 2020 [51]	200	400	80	30
45	98.8	LOF	Huang. et al., 2020 [56]	300	600	80	30
46	83	LOF	Trevisan et al., 2017 [57]	195	400	80	30

## References

[1] Tang M., Pistorius P.C., Beuth J.L. (2017). Prediction of Lack-of-Fusion Porosity for Powder Bed Fusion. Addit. Manuf..

[2] Leis A., Weber R., Graf T. (2021). Process Window for Highly Efficient Laser-Based Powder Bed Fusion of AlSi10Mg with Reduced Pore Formation. Materials.

[3] Foteinopoulos P., Papacharalampopoulos A., Angelopoulos K., Stavropoulos P. (2020). Development of a Simulation Approach for Laser Powder Bed Fusion Based on Scanning Strategy Selection. Int. J. Adv. Manuf. Technol..

[4] Sadeghilaridjani M., Ladani L. (2022). Location-Dependent Deformation Behavior of Additively Manufactured Copper and Copper-Carbon Nanotube Composite. J. Alloys Compd..

[5] Martucci A., Aversa A., Lombardi M. (2023). Ongoing Challenges of Laser-Based Powder Bed Fusion Processing of Al Alloys and Potential Solutions from the Literature—A Review. Materials.

[6] Thangamani G., Felicioni S., Padovano E., Biamino S., Lombardi M., Ugues D., Fino P., Bondioli F. (2024). A Comprehensive Review of Laser Powder Bed Fusion in Jewelry: Technologies, Materials, and Post-Processing with Future Perspective. Metals.

[7] Sadowski M., Ladani L., Brindley W., Romano J. (2016). Optimizing Quality of Additively Manufactured Inconel 718 Using Powder Bed Laser Melting Process. Addit. Manuf..

[8] Hassanin H., El-Sayed M.A., Ahmadein M., Alsaleh N.A., Ataya S., Ahmed M.M.Z., Essa K. (2023). Optimising Surface Roughness and Density in Titanium Fabrication via Laser Powder Bed Fusion. Micromachines.

[9] Agrawal A.K., Rankouhi B., Thoma D.J. (2022). Predictive Process Mapping for Laser Powder Bed Fusion: A Review of Existing Analytical Solutions. Curr. Opin. Solid State Mater. Sci..

[10] Mukherjee T., DebRoy T. (2018). Mitigation of Lack of Fusion Defects in Powder Bed Fusion Additive Manufacturing. J. Manuf. Process..

[11] Johnson L., Mahmoudi M., Zhang B., Seede R., Huang X., Maier J.T., Maier H.J., Karaman I., Elwany A., Arróyave R. (2019). Assessing Printability Maps in Additive Manufacturing of Metal Alloys. Acta Mater..

[12] Romano J., Ladani L., Sadowski M. (2016). Laser Additive Melting and Solidification of Inconel 718: Finite Element Simulation and Experiment. JOM.

[13] Romano J., Ladani L., Sadowski M. (2015). Thermal Modeling of Laser Based Additive Manufacturing Processes within Common Materials. Procedia Manuf..

[14] Foteinopoulos P., Papacharalampopoulos A., Stavropoulos P. (2024). Additive Manufacturing Simulations: An Approach Based on Space Partitioning and Dynamic 3D Mesh Adaptation. Addit. Manuf. Lett..

[15] Ladani L. (2021). Additive Manufacturing of Metals: Materials, Processes, Tests, and Standards.

[16] Rubenchik A.M., King W.E., Wu S.S. (2018). Scaling Laws for the Additive Manufacturing. J. Mater. Process. Technol..

[17] Jadhav S.D., Goossens L.R., Kinds Y., Van Hooreweder B., Vanmeensel K. (2021). Laser-Based Powder Bed Fusion Additive Manufacturing of Pure Copper. Addit. Manuf..

[18] Yadroitsev I., Gusarov A., Yadroitsava I., Smurov I. (2010). Single Track Formation in Selective Laser Melting of Metal Powders. J. Mater. Process. Technol..

[19] King W.E., Barth H.D., Castillo V.M., Gallegos G.F., Gibbs J.W., Hahn D.E., Kamath C., Rubenchik A.M. (2014). Observation of Keyhole-Mode Laser Melting in Laser Powder-Bed Fusion Additive Manufacturing. J. Mater. Process. Technol..

[20] Rosenthal D. (1941). Mathematical Theory of Heat Distribution during Welding and Cutting. Weld. J..

[21] Richter B., Pribe J.D., Weber G.R., Subraveti V., Oskay C. (2025). Analytical Prediction of Lack-of-Fusion Porosity Including Uncertainty and Variable Melt Pools for Powder Bed Fusion. Addit. Manuf..

[22] Ho V., Ladani L., Razmi J., Gruber S., Murphy A.B., Chen C., East D., Lopez E. (2025). Powder Bed Fabrication of Copper: A Comprehensive Literature Review. Metals.

[23] Ladani L., Sadeghilaridjani M. (2021). Review of Powder Bed Fusion Additive Manufacturing for Metals. Metals.

[24] Seidgazov R.D. (2009). Thermocapillary Mechanism of Melt Displacement during Keyhole Formation by the Laser Beam. J. Phys. D Appl. Phys..

[25] Imani Shahabad S., Karimi G., Toyserkani E. (2021). An Extended Rosenthal’s Model for Laser Powder-Bed Fusion Additive Manufacturing: Energy Auditing of Thermal Boundary Conditions. Lasers Manuf. Mater. Process..

[26] Keeley W., Turner R., Mitchell B., Warnken N. (2025). A Development of the Rosenthal Equation for Predicting Thermal Profiles During Additive Manufacturing. Thermo.

[27] Wei H.L., Mukherjee T., Zhang W., Zuback J.S., Knapp G.L., De A., DebRoy T. (2021). Mechanistic Models for Additive Manufacturing of Metallic Components. Prog. Mater. Sci..

[28] Nordet G., Gorny C., Mayi Y., Daligault J., Dal M., Effernelli A., Blanchet E., Coste F., Peyre P. (2022). Absorptivity Measurements during Laser Powder Bed Fusion of Pure Copper with a 1 kW Cw Green Laser. Opt. Laser Technol..

[29] Rosenthal D. (1946). The Theory of Moving Sources of Heat and Its Application to Metal Treatments. J. Fluids Eng..

[30] Bergman T.L., Lavine A.S. (2017). Fundamentals of Heat and Mass Transfer.

[31] Qu S., Ding J., Fu J., Fu M., Zhang B., Song X. (2021). High-Precision Laser Powder Bed Fusion Processing of Pure Copper. Addit. Manuf..

[32] TRUMPF (2025). Parameter Set TruPrint 1000 Copper. TRUMPF AM Datasheet Copper TruPrint 1000.

[33] Robinson J., Arjunan A., Baroutaji A., Stanford M. (2021). Mechanical and Thermal Performance of Additively Manufactured Copper, Silver and Copper–Silver Alloys. Proc. Inst. Mech. Eng. Part L J. Mater. Des. Appl..

[34] Bonesso M., Rebesan P., Gennari C., Mancin S., Dima R., Pepato A., Calliari I. (2021). Effect of Particle Size Distribution on Laser Powder Bed Fusion Manufacturability of Copper. BHM Berg. Hüttenmänn. Monatshefte.

[35] Domine A., Verdy C., Penaud C., Vitu L., Fenineche N., Dembinski L. (2023). Selective Laser Melting (SLM) of Pure Copper Using 515-Nm Green Laser: From Single Track Analysis to Mechanical and Electrical Characterization. Int. J. Adv. Manuf. Technol..

[36] Lassègue P., Salvan C., De Vito E., Soulas R., Herbin M., Hemberg A., Godfroid T., Baffie T., Roux G. (2021). Laser Powder Bed Fusion (L-PBF) of Cu and CuCrZr Parts: Influence of an Absorptive Physical Vapor Deposition (PVD) Coating on the Printing Process. Addit. Manuf..

[37] Gruber S., Stepien L., López E., Brueckner F., Leyens C. (2021). Physical and Geometrical Properties of Additively Manufactured Pure Copper Samples Using a Green Laser Source. Materials.

[38] SLM Solutions (2024). Preparing Your Process and Facility for Metal Additive Manufacturing.

[39] Azo Materials H13 Tool Steel—Chromium Hot-Work Steels. https://www.azom.com/article.aspx?ArticleID=9107.

[40] Xometry Pro Aluminium AlSi10Mg. Technical Datasheet. https://xometry.pro/wp-content/uploads/2023/08/Aluminium-1706-1.pdf.

[41] ASM Aerospace Specification Metals Inc Titanium Ti-6Al-4V (Grade 5), STA. https://www.aerospacemetals.com/wp-content/uploads/2023/07/Titanium-Ti-6Al-4V-Grade-5-STA-Data-Sheet.pdf.

[42] Brandau B., Da Silva A., Wilsnack C., Brueckner F., Kaplan A.F.H. (2022). Absorbance Study of Powder Conditions for Laser Additive Manufacturing. Mater. Des..

[43] Yan J., Zhou Y., Gu R., Zhang X., Quach W.-M., Yan M. (2019). A Comprehensive Study of Steel Powders (316L, H13, P20 and 18Ni300) for Their Selective Laser Melting Additive Manufacturing. Metals.

[44] Honda H., Watanabe M. (2024). Quantifying Laser Absorptivity of Ti–6Al–4V Powder through Additive Manufacturing Systems. Mater. Trans..

[45] Miyagi M., Zhang X. (2015). Investigation of Laser Welding Phenomena of Pure Copper by X-Ray Observation System. J. Laser Appl..

[46] Aboulkhair N.T., Simonelli M., Parry L., Ashcroft I., Tuck C., Hague R. (2019). 3D Printing of Aluminium Alloys: Additive Manufacturing of Aluminium Alloys Using Selective Laser Melting. Prog. Mater. Sci..

[47] He Y., Zhong M., Beuth J., Webler B. (2020). A Study of Microstructure and Cracking Behavior of H13 Tool Steel Produced by Laser Powder Bed Fusion Using Single-Tracks, Multi-Track Pads, and 3D Cubes. J. Mater. Process. Technol..

[48] He Y., Montgomery C., Beuth J., Webler B. (2019). Melt Pool Geometry and Microstructure of Ti6Al4V with B Additions Processed by Selective Laser Melting Additive Manufacturing. Mater. Des..

[49] Hooper P.A. (2018). Melt Pool Temperature and Cooling Rates in Laser Powder Bed Fusion. Addit. Manuf..

[50] Williams R.J., Piglione A., Rønneberg T., Jones C., Pham M.-S., Davies C.M., Hooper P.A. (2019). In Situ Thermography for Laser Powder Bed Fusion: Effects of Layer Temperature on Porosity, Microstructure and Mechanical Properties. Addit. Manuf..

[51] Yan X., Chang C., Dong D., Gao S., Ma W., Liu M., Liao H., Yin S. (2020). Microstructure and Mechanical Properties of Pure Copper Manufactured by Selective Laser Melting. Mater. Sci. Eng. A.

[52] Constantin L., Wu Z., Li N., Fan L., Silvain J.-F., Lu Y.F. (2020). Laser 3D Printing of Complex Copper Structures. Addit. Manuf..

[53] Malý M., Koutný D., Pantělejev L., Pambaguian L., Paloušek D. (2022). Effect of High-Temperature Preheating on Pure Copper Thick-Walled Samples Processed by Laser Powder Bed Fusion. J. Manuf. Process..

[54] Jadhav S.D., Dadbakhsh S., Goossens L., Kruth J.-P., Van Humbeeck J., Vanmeensel K. (2019). Influence of Selective Laser Melting Process Parameters on Texture Evolution in Pure Copper. J. Mater. Process. Technol..

[55] Jadhav S.D., Vleugels J., Kruth J., Van Humbeeck J., Vanmeensel K. (2020). Mechanical and Electrical Properties of Selective Laser-melted Parts Produced from Surface-oxidized Copper Powder. Mater. Des. Process. Commun..

[56] Huang J., Yan X., Chang C., Xie Y., Ma W., Huang R., Zhao R., Li S., Liu M., Liao H. (2020). Pure Copper Components Fabricated by Cold Spray (CS) and Selective Laser Melting (SLM) Technology. Surf. Coat. Technol..

[57] Trevisan F., Calignano F., Lorusso M., Lombardi M., Manfredi D., Fino P. Selective Laser Melting of Chemical Pure Copper. Proceedings of the Euro PM 2017: International Powder Metallurgy Congress and Exhibition.

[58] Sinico M., Cogo G., Benettoni M., Calliari I., Pepato A. Influence of powder particle size distribution on the printability of pure copper for selective laser melting. Proceedings of the 30th Annual International Solid Freeform Fabrication Symposium—An Additive Manufacturing Conference.

[59] Tang C., Le K.Q., Wong C.H. (2020). Physics of Humping Formation in Laser Powder Bed Fusion. Int. J. Heat Mass Transf..

[60] Khairallah S.A., Anderson A.T., Rubenchik A., King W.E. (2016). Laser Powder-Bed Fusion Additive Manufacturing: Physics of Complex Melt Flow and Formation Mechanisms of Pores, Spatter, and Denudation Zones. Acta Mater..

[61] Imai K., Ikeshoji T.-T., Sugitani Y., Kyogoku H. (2020). Densification of Pure Copper by Selective Laser Melting Process. Mech. Eng. J..

[62] Demir A.G., Colopi M., Previtali B. The Use of a Ns-Pulsed, High Repetition Rate Green Laser for SLM of 99.9% Pure Cu. Proceedings of the 2019 Lasers in Manufacturing Conference (LiM).

[63] Corona D., Giannini O., Guarino S., Ponticelli G.S., Zarcone M. (2022). Experimental Investigation on the Electrical, Thermal, and Mechanical Properties of Laser Powder Bed Fused Copper Alloys. J. Manuf. Process..

[64] Guan J., Zhang X., Jiang Y., Yan Y. (2019). Insights into Fabrication Mechanism of Pure Copper Thin Wall Components by Selective Infrared Laser Melting. Rapid Prototyp. J..

